# Berlin Notes

**Published:** 1874-06-01

**Authors:** M. W. Hatfield


### Correspondence.

### BERLIN NOTES.—RED TAPES.

*By M. W. Hatfield, M.D.*

IF you can spare a winter for attendance on the Berlin hospitals, by all means avail yourself of their unsurpassed facilities for study; but when you go, don't forget to leave your American impatience at home. Time seems to be nearly valueless in Berlin and, unless you cultivate German imperturbability there, you will probably keep yourself in a constant fret over the vexatious delays which will meet you at every turn. These usually begin with the two weeks enforced waiting which greets the student who has strained every nerve to arrive at the time officially announced for the commencement of the session. It may be that he is unreasonable enough to fail to see why the professors ought to have an additional fortnight of grace from both ends of the term, but custom gives it them and you may employ the time in grumbling, or more profitably in learning the mazes of the charité hospital and university red tape.

And first you will learn that matriculation into a K. K. institution is not to be lightly despatched in half a dozen minutes. It is a time-honored ceremonial, which must be soberly and advisedly performed, as follows: A many-buttoned official demands your name and passport at the outer door. If these are satisfactory, you are ushered into the grand hall, which is well filled with students—of law, theology, medicine and philosophy—all of whom must properly matriculate before the grave and revered professors who sit clustered about a long table. Around this the candidates pass, answering questions as they go, and for an hour it is amusing enough to watch their behavior during this catechism by the *geheimrathen*. Students come and students go, and at last you hear the German equivalent for your name and are pushed hurriedly forward to the table. Prof. No. 1 takes your passport from a pile of documents, identifies you from it, files it away among the archives of the university and then passes you on to the gentleman on his right. He continues the examination concerning previous studies, etc., writes a short history of your life in a ponderous book and finally recommends you to the rector as a *vir juvenis ornatissimus* and suitable for matriculation. The venerable rector inscribes your name upon a formidable diploma which informs the world in the choicest Latin that by the grace of God and the King of Prussia your name is now entered among those who have legitimately matriculated at the Frederick William University. This document entitles you to the attention of the next professor, who kindly invites you to register in the appropriate columns of another ponderous book the most important facts in your life and your parents', and after another cross-examination, you pass to the treasurer. He graciously allows you to drop the matriculation fee into his strong box and in return presents a handful of pamphlets and a student's card, which protects from municipal arrest in case of drunkenness or other misdemeanors—though in such cases there is a trial by the University Gericht, which would probably not be a change for the better. These, and other interesting facts, can be gleaned from the 28 pages of laws just received, as you sit waiting the course of events, for the end is not yet. After about twenty more students have received their cards, the rector deems his audience large enough to listen to a "few remarks," so arises, puts on his cap of office, and coming to the front of the table, clutches it with one hand and saws the air with the other as he delivers a set speech upon freedoms and the inestimable privilege of being a student in the Royal University of Berlin. This done, he offers his right hand of fellowship to the students, who squeeze it à la Presidential levee as they pass by in Indian file. Then the others depart, the "medics" are still obliged to wait the coming of the Dean of the Medical Department. A quarter and a half an hour pass in this way, and just as patience is ceasing to be a virtue, a Charles-Darwin-ish-looking man enters the room and the students crowd about his desk with their usual courtesy, compelling the strangers to wait another good half hour before they can approach Herr Bardelebein. There the previous questions concerning age, studies, etc., must again be answered and recorded in another ponderous book, another diploma, more schedules, a library card, etc., are presented, and thus for the present ends matriculation proper.

All this amounts to, however, is simply two weeks admission to the lectures, if you cannot decide in less time which you wish to attend. Sooner or later you again provide yourselves with the needful Frederick d'ors, (half for each professor,) note in your anmeldungsbuch the lectures you have selected and search out the grantor's office. Step softly and bow low as you enter into the presence of this awful specimen of a petty official, "dressed in a little brief authority," etc. On the morning we visited him he happened to be in one of his worst humors and consequently growls away savagely at some slight irregularity in our books, favors us with his ideas on Americans remaining at home and other pleasing topics, but at length is condescending enough to receive our lecture fees—and fifteen cents for himself—and gives us in return a number of printed receipts, which we must hand over to the respective professors named therein, and receive in return cards admitting us regularly to their lectures. Hence a week's hide and go seek with the above mentioned gentleman, of which Prof. Martin requires two days, for the first time he is caught at ballottement and requires a second call at his office the next day. Prof. Hanock kindly interrupts a children's clinic to give us the desired cards. Baron Von Langenbeck is at last found in the otium cum dig. of a post-prandial cigar, while Prof. Reichert is discovered raging to and fro through his magnificent dissecting rooms. Herr Geheimrath Frerichs is "balmy" and consequently gives us the best seats in his room, but Vierchow is as dry and crusty as his beloved specimens, for one of which he could easily be mistaken.

Well, thank fortune, we are at last ready to begin work ; but you are by no means through with the red tape, whose turns embrace about everything in Berlin. For instance, you wish to procure a book from the great library and hasten there with the library card that you received on matriculation. You present it, and learn that it only entitles you to draw books from the University Library, which, by the way, no student was ever known to use. This is the Royal K. K. Library, and to use it you must present a certificate of moral character, etc. So you hunt up a professor and obtain from him his signature to a printed promise that he will be personally responsible for all books you fail to bring back, and with this, on the morrow, hasten triumphantly back for the book. Not quite yet, if you please, but if you come to-morrow at noon you may have a card and the printed forms necessary to obtain books. You do so, and after registration and cross-examination are told to fill up one of the blanks, deposit it in a letter box, and by calling at a very inconvenient hour the next day you can at last obtain the desired book. And this is only one of the many beauties of Prussian "system," which encompasses you on every hand. Simply the admission to a student's mutual aid society requires more registration than would be necessary to buy a house and lot at home. Change your residence and there must be notice of the same given both to the university and nearest police office, and woe unto you if you do not correctly answer all the questions that the curiosity of the polizisamt can ask, for we wot of two students that came to grief from failing to classify their religion according to the Prussian nomenclature. And last, but not least, you will receive some fine morning an invitation from the Prussian Government to please step up to the office and pay a tax on their estimate of your income. Perhaps you fail to see the justice of this and you apply to the American Ambassador, who consoles you by telling you that it needs must be, and you may rest assured that the Prussian officials will not forget you. Nor do they, for after a brisk correspondence they will name a day of wrath, on which, if your tax is not paid, your personal property will be sold to the highest bidder, and as a friend we recommend that the tax be paid or that you move before that date to fairer climes. If you are a student you may not find this so easy, for the powers that be have your passport and to obtain it you must return your anmeldungbuch, properly signed by all the professors, whose lectures you have attended during the past term. Also a certificate from the university library to the effect that you have taken out no books and returned them all safely, etc., and in process of time—ordinarily about a week—you will receive your passport and a dismissal, without which you can enter no other German university. There is, however, one other way in which you can regain your passport, and that is by applying for a permission to travel from the Gericht, and if your reasons seem good, you may obtain a Reisescheim for a certain specified time, and this, when the King's business requires haste, is certainly the most expeditious way of saying Good Bye to Berlin, though you must be back at the appointed time or be black-boarded.

